# Supplementary figures and images for: Larval ecology and bionomics of Anopheles funestus in highland and lowland sites in western Kenya
Source: PLoS One. 2021 Oct 11;16(10):e0255321. doi: 10.1371/journal.pone.0255321 (PMC8504749; doi:10.1371/journal.pone.0255321)

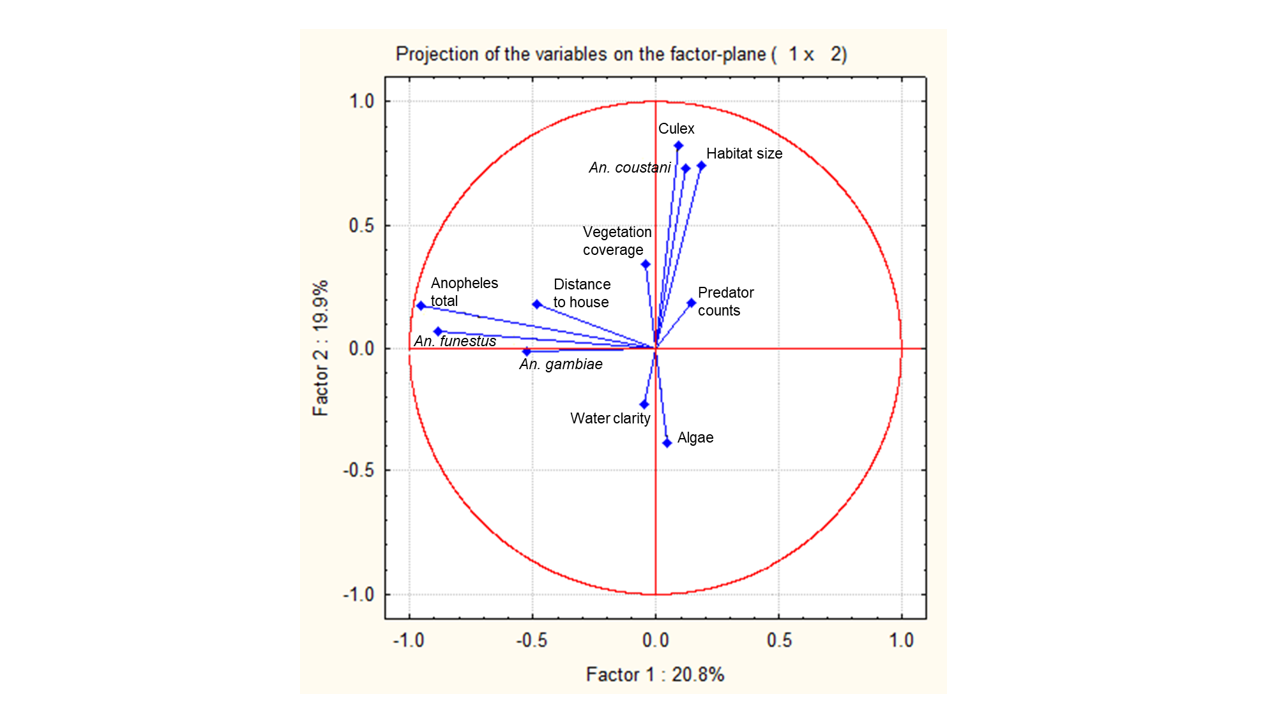

Supplement: S1 Fig — (TIF) [file pone.0255321.s001.tif]
